# Observational Studies in Male Elite Football: A Systematic Mixed Study Review

**DOI:** 10.3389/fpsyg.2019.02077

**Published:** 2019-10-18

**Authors:** Maria Preciado, M. Teresa Anguera, Mauricio Olarte, Daniel Lapresa

**Affiliations:** ^1^Department of Social Psychology and Quantitative Psychology, Faculty of Psychology, University of Barcelona, Barcelona, Spain; ^2^Faculty of Psychology, Institute of Neurosciences, University of Barcelona, Barcelona, Spain; ^3^National Administrative Department of Statistics, Bogotá, Colombia; ^4^Department of Educational Sciences, University of La Rioja, Logroño, Spain

**Keywords:** professional soccer, mixed methods, systematic observation, methodological systematic review, GREOM

## Abstract

**Objective:** This systematic mixed study review, focuses on the use of observation methodology in elite men's football matches, which constitutes an innovative approach, that opens up a new panorama of useful and productive research.

**Method:** The methods used in this study follow the recommendations for systematic reviews and meta-analysis guidelines (PRISMA). The search was carried out in five databases. Ninety-four articles out of 3,195 were selected and analyzed. In order to achieve a quality assessment, the guide was used to inform evaluations based on observation methodology (GREOM) (Portell et al., 2015), recognized by the EQUATOR network.

**Results:** From the methodological review analysis, information obtained indicates that 97% of the researches used direct observation and 3% indirect observation. On the other hand, 56.5% of the articles explain the instrument used and 77% justify the applied observational design. A quantitative comparison of the proportions was made in several methodological aspects, which resulted in only 15.21% reviewing the quality of the data, and that 67.3% of the articles contributed to the mixed methods approach. The methodological review allowed us to establish procedural profiles. The results indicate that 67% of the articles have been published in English, and of these, 77% were published in journals that have an impact factor. The majority of the researchers, 53.26%, belong to Spanish entities. The most studied substantive aspects were goal (34%), possession of the ball (28%), and corner (27%). The most observed events were Leagues, World Cups, individual players and other events.

**Conclusions:** The results obtained refer to both substantive and methodological aspects and allow us to configure a systematic review of mixed studies, in which we emphasize the aspects of a “systematic review” and a “mixed study,” within an integrated perspective.

## Introduction

Currently there is a growing interest in the realization of studies that evaluate the quality of research, combining conceptual, and methodological reviews. We know that systematic reviews present methodological conflicts when the *leitmotiv* focuses on a procedural interest. Despite that fact, there have been precedents in which the selection criteria for primary studies were carried out based on the combination of a methodological criterion and another noun (López-Fernández et al., 2017) or by considering procedural criteria, such as units of analysis or operationalization of constructs (Durach et al., 2017) or by focusing on terminological matters (Logan et al., 2018). We consider it an enrichment for the *systematic mixed study review* itself, and in our case, the vertebrate element consists of the combination of a methodological criterion, which is observational methodology, and a substantive criterion, which is elite male football; all inscribed in a *mixed methods* perspective.

According to Hong and Pluye (2018, p. 1), “Systematic reviews are considered among the best sources of research evidence, are used for decision making, and are helpful in coping with the rapidly increasing volume of scientific literature.” Now, if we specify more, “we (…) define a systematic review as a review of existing research using explicit accountable rigorous research methods” (Gough et al., 2012, p. 2) or, in more detail, “a systematic review is a review of a clearly formulated question that uses systematic and explicit methods to identify, select, and critically appraise relevant research, and to collect and analyze data from the studies that are included in the review” (Moher et al., 2009, p. 265).

The general claim of *systematic reviews* has been to obtain a comprehensive synthesis of evidence (Higgins and Green, 2011) about a space of knowledge in which various publications have been generated (Grant and Booth, 2009; Boland et al., 2013; Maden and Kotas, 2016). The “space of knowledge,” in this work, is procedural, which represents a new line of research, given that it will provide all the necessary elements to assess the strengths and weaknesses of research in mixed methodological aspects as well as in the substantive. We have taken a step forward, entering the *mixed methods* perspective, and according to Pluye and Hong (2014), mixed study reviews integrate qualitative, quantitative and mixed methods perspectives. These authors, in turn, define mixed methods as: *a research approach in which a researcher or team of researchers integrates (a) qualitative and quantitative research questions, (b) qualitative research methods and quantitative research designs, (c) techniques for collecting and analyzing qualitative and quantitative data, and (d) qualitative findings and quantitative results* (Pluye and Hong, 2014, p, 29).

Sandelowski et al. (2006), as well as Heyvaert et al. (2013), concur with Pluye and Hong (2014) in proposing the confluence of QUAN (quantitative), QUAL (qualitative), and MM (mixed methods) articles in systematic reviews. Each of them responds to its methodological approach, although here we are particularly interested in the focus on mixed methods. At present, *systematic mixed study reviews* have been proposed to provide an answer to a well-defined substantive question that internalizes an integration between qualitative and quantitative elements (Moseholm and Fetters, 2017).

*Mixed methods* can be conceptualized from epistemological debates between supporters of qualitative and quantitative aspects (Pluye and Hong, 2014), and it is required—and we advise—to use them with the utmost rigor, considering their differentiation from the *multimethods* approach (Anguera et al., 2018b), which has not always been clear. Guaranteeing integration and qualitative-quantitative symmetry (Anguera et al., 2017a), are questions that remain unresolved.

Male football, without contemplating a methodological plus, has been subject to other systematic reviews (Sarmento et al., 2014, 2018; Trewin et al., 2017; McGuckian et al., 2018), and is also an elite category in various sports (Spindler et al., 2018).

Performance in football is a polyhedral issue, a product of the dynamic interaction between competitors through game actions consisting of different complementary facets; physical, technical, tactical, etc. (Barbosa et al., 2013; Liu et al., 2016). In recent years we have witnessed a vertiginous evolution in the *match analysis*, mainly motivated by the emergence of automatic registration procedures, which allows the immediate acquisition of a large amount of data related to the positioning of the players in relation to the game (Castellano et al., 2014). However, these data, obtained through a *computerized video tracking system*, need to be enriched with other technical-tactical data, recorded semi-automatically through visualization, recording, and coding (Hernández-Mendo et al., 2014).

This evolution could not remain indifferent to the approach of mixed methods studies, which during the last two decades have overcome a traditional procedural confrontation that has affected all scientific fields, and consequently, also studies on sport (Anguera et al., 2017b) and therefore, on professional football. Johnson et al. (2007, p. 123) defined mixed methods research as “the type of research in which a researcher or team of researchers combines elements of qualitative and quantitative research approaches (e.g., use of qualitative and quantitative viewpoints, data collection, analysis, inference techniques) for the broad purposes of breadth and depth of understanding and corroboration” (Johnson et al., 2007, p. 123). This new methodological positioning has required a deep reflection on the key concepts of mixed methods and has promoted the articulation of ways in which to integrate qualitative and quantitative elements.

In the search for a rigorous and flexible methodological framework which allows the possibility of capturing the behaviors that unfold over time (Bakeman and Quera, 2011) and the consequent performance of diachronic analysis that allow the detection of regular behavioral structures we find that many of the studies analyzed have resorted to observational methodology (Anguera, 1979; Anguera et al., 2017b), and there is ample evidence over the last years in football (Jonsson et al., 2006; Castellano et al., 2007; Lapresa et al., 2013, 2015, 2016, 2018). This extensive scientific production in football, conducted from observational methodology, is based precisely on the need for investigations to adapt to *mixed methods*.

Currently, the observational methodology considered to be mixed methods in itself, has established that in the same study the stages QUAL-QUAN-QUAL are complemented, according to the *connect* path recommended by Creswell and Plano Clark (2011) in addition to the *merged* and *embed* paths. We consider that this approach is optimal, given that it starts (QUAL stage) with a qualitative collection of data, which can initially be descriptive, but will be systematized, especially when it is already coded from the *ad hoc* prepared observation instrument. This provides all the information collected in a matrix of codes, with columns as the dimensions/sub-dimensions of the observation instrument, and rows as the contemplated observed units. This is then segmented according to the established criteria in each case. In a second stage (QUAN stage), the quality control of the data is carried out and once passed, a quantitative data analysis is carried out. This provides the results of the study, which, in a third stage (QUAL stage) is interpreted in light of the scientific problem posed and the results are then obtained by reference authors of the question studied.

From this proposal by Creswell and Plano Clark (2011), we highlight the relevant role of *connect*, that reveals the value of the transformative capacity that the data facilitates for the intended integration (O'Cathain et al., 2010), which in recent years had promoted conceptual and procedural advances.

As indicated by Anguera and Hernández-Mendo (2014), at the dawn of Sports Psychology there was hardly any observation known as a research methodology (Tod et al., 2010). It was Martens (1979), on the one hand, and Smith et al. (1977), on the other, who respectively proposed the use of observation in the field of sport and proposed an initial observation instrument.

Biddle (1997), who in a study in the most prestigious journals of the time—*The Journal of Sport and Exercise Psychology* (ISEP) and *The International Journal of Sport Psychology* (IJSP)—realized that during the nineties, most quantitative sports studies were based on regression and discriminant analysis techniques, and most qualitative sports studies focused on interviewing and content analysis. And Morris (1999) reported that observational studies (in addition to case studies) accounted for 2% of scientific production in this field between 1979 and 1998. Consequently, the use of observation in sport was negligible.

Bakeman and Gottman (1997, p. 3) define systematic observation “as a particular approach to quantifying behavior,” that marks an issue of exceptional transcendence and that we have always had in mind. In our research group numerous works have been published (Anguera, 1979, 2003; Anguera et al., 2001, 2017a, 2018a; Blanco-Villaseñor et al., 2003; Sánchez-Algarra and Anguera, 2013; Portell et al., 2015), over several decades, which have contributed to the consolidation of the observational methodology. It is a “new” way of carrying out scientific studies in situations in which both the naturalness of the situation and spontaneous behavior are respected. In the eighties and nineties more works were published that are considered precursors of systematic observation, and in the sports field, and, specifically, in professional football, it has had high applicability.

In Anguera et al. (2017b), one more step is taken, justifying the inclusion of purely observational studies of sport and physical activity as *mixed methods*, and we did various works that go in this direction (Camerino et al., 2012b; Anguera et al., 2014; Anguera and Hernández-Mendo, 2016), establishing that observational methodology can be considered a *mixed method* in itself and therefore, through successive stages combines qualitative and quantitative elements.

Observational research applied to the study of sport, starting in the first decade of this second millennium, has been progressively structured on solid methodological foundations that we can consider consolidated today, in regards to observational designs (Anguera et al., 2011), and the record's systematization and coding (Anguera and Blanco-Villaseñor, 2003). In addition, in recent years, work has been done not only on the consolidation of direct observation (Anguera, 2003; Sánchez-Algarra and Anguera, 2013; Portell et al., 2015; Anguera et al., 2017b), but in the conceptualization and development of indirect observation (Anguera et al., 2018c), which can be very relevant in the study of different aspects of professional football.

A recent milestone (July 2017) of great importance, highlighting the relevance of observational methodology, is the publication of *guidelines* for the evaluation of the effectiveness in studies based on observational methodology, that can be found on the EQUATOR Network [*http://www.equator-network.org/reporting-guidelines/guidelines-for-reporting-evaluations-based-on-observational-methodology/]*, and which are open to the international scientific community. Highlighting the incidence of systematic observation in scientific studies of sport—which of course includes football—it is equally relevant that in 2016 a Research Topic was launched in the journal *Frontiers in Psychology*, with high visibility, entitled *Systematic observation: Engaging researchers in the study of daily life as it is lived* [*http://journal.frontiersin.org/researchtopic/4846/systematic-observation-engaging-researchers-in-the-study-of-daily-life-as-it-is-lived]*, which has intensified the use of systematic observation, and in 2018 the same scientific journal initiated another Research Topic entitled *Best Practice Approaches for Mixed Methods Research in Psychological Science* [*https://research-topic-management-app.frontiersin.org/manage/8170/dashboard]*, in which a good number of articles consider, already previously justified in Anguera and Hernández-Mendo (2016) and Anguera et al. (2017b), that observational methodology could be regarded as *mixed methods* in itself. We hope that they will follow the advances mentioned in *synthetizing* (Anguera and Hernández-Mendo, 2019). In this work we adopt the assertion, that the observational methodology could be considered as *mixed methods* in itself as *leitmotiv*.

Currently, experience of this approach has been gained aiding its application through indirect observation and now in the field of sports (García-Fariña et al., 2018), which initially only appeared as possible in direct observation (Anguera et al., 2017b, 2018c; Anguera, 2019, 2020).

The interest in the integration between qualitative and quantitative elements is the *leitmotiv* that in recent years most characterize authors who are positioned in the *mixed methods* approach, and this influence shows a growing maturity, which is progressively being implemented in scientific literature (Onwuegbuzie et al., 2009; O'Cathain et al., 2010; Moseholm and Fetters, 2017).

The objective of this systematic mixed study review focuses on the use of observational methodology without neglecting the substantive in elite male football matches, constituting an innovative approach that opens up a new useful and productive research landscape.

This unfolding interest for the new advances in mixed methods allows us to argue that this study on the systematic mixed study review focuses on the procedural aspect of systematic observation, although it is substantively situated in studies carried out on elite football.

## Methods

### Procedure

According to the recommendations of the elements of the *Preferred Reporting Items for Systematic reviews and Meta analyses Guidelines* (PRISMA) to present the findings of systematic reviews (Liberati et al., 2009), the search was carried out in electronic databases; these databases included the web of science, Scopus, ProQuest, Google Scholar, Scielo, and Dianet plus. The topic focused on observational research in elite football. The temporary location of the bibliographic references corresponds to the period from January 1996 to December 2018. The descriptors used were football, soccer, men, analysis, observation, defense, match analysis, performance analysis, notational analysis, game analysis, tactical analysis, and game patterns.

The inclusion criteria were as follows: empirical articles in Spanish, English, and Portuguese, published in any country, in the indicated period that contained some of the descriptors, specification of the objective, and guarantee that they had a minimum of quality in accordance with the selected GREOM scale (Portell et al., 2015), and also the checklist to measure the quality of the reporting of sports-related observational studies (Chacón-Moscoso et al., 2018). Exclusion criteria included: other sports, women, children, other research and population methods, coaches, and goalkeepers. The review process was carried out with the keywords and the Boolean operator AND, OR, NOT. Ninety-four articles of a total of 3,197 were selected and analyzed through a systematic mixed methods review.

The search procedure was carried out simultaneously by two of the main investigators, then the articles were selected by relevance of title and objective topic, continuing with the reading of the abstracts by those responsible for the search. The researchers compared the articles, grouping them into categories according to the minimum quality of the GREOM, then analyzed the differences and discussed those that presented some doubts. Finally, once the final selection was made, a complete reading of the selected documents was submitted, subject to the approval of both researchers. When doubts had no solution, a third party was consulted to define the selections; this entire procedure was carried out to avoid the risk of bias.

**Figure 1:**
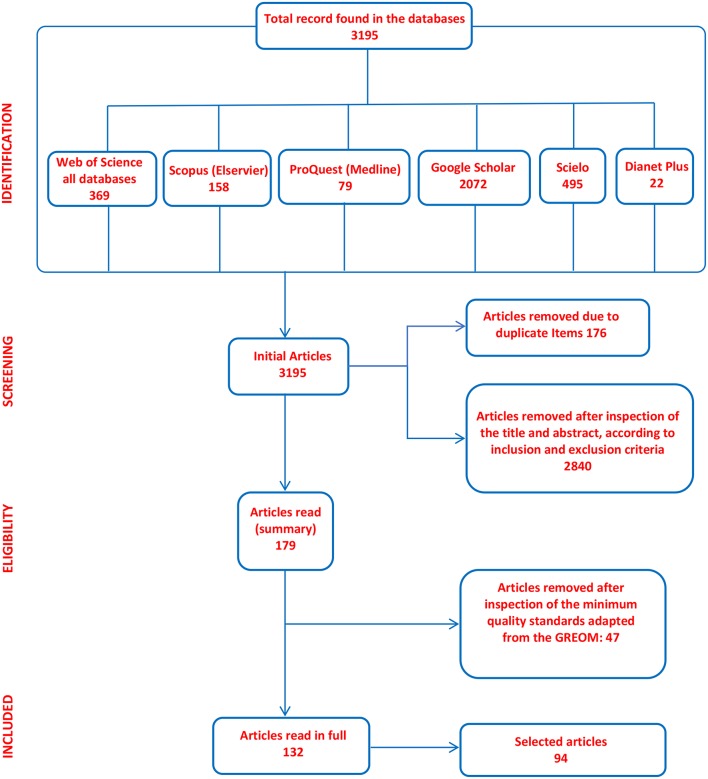
Selection process of primary documents.

The process of selecting the primary documents is shown in the following PRISMA flow diagram (Figure 1).

### Issues for Methodological Review

We have referred to the GREOM guide (Portell et al., 2015), which provides consistency and strength to the scientific logic of observational studies, when considering the following aspects:

#### Direct/Indirect Observation

Within the framework of Domain A of the GREOM, and the result of the conceptualization that has been carried out over the last few years, the difference between direct (Sánchez-Algarra and Anguera, 2013) and indirect observation (Anguera et al., 2018c; Anguera, 2019) has been clarified, which basically corresponds, respectively, to total or partial perceptivity. Direct observation is considered a situation in which the starting point is the recording of the game sessions and is, therefore, derived from visually perceptible information. On the contrary, works in which documentary, tabular or graphic information from a sports institution or companies that make these data bases, were considered as an indirect observation. This differentiation should barely affect the development of the stages of the procedure.

#### Observational Design

Domain B of the GREOM specifies the relevance of the observational design, which acts as a guideline for the researcher conducting the study. The concept of observational designs (Anguera et al., 2001, 2011) has contributed to consistency in the use of observation in sports (Anguera and Hernández-Mendo, 2014). A new flexible structure has been configured that channels scientific procedure from perceptible reality data, and a clear congruence has been fostered in the construction of instruments, in the decision about the data, and in possible subsequent data analyses.

#### Observation and Recording Instruments

Both instrument modalities are required in systematic observation studies. Bearing in mind that the observation instrument is of a non-standard nature, and has been tailor-made (*ad hoc*), following the GREOM guidelines its modality is specified (category system, field format, combination of field format and category system, or *rating scales*) (Anguera et al., 2007; Sánchez-Algarra and Anguera, 2013). It also indicates what recording instrument, a computer program (Hernández-Mendo et al., 2014), was used to carry out the recording and/or, where appropriate, what electronic device was used (such as tracking systems, and others).

#### Record Parameters

There are many types of records, from descriptive or non-systematized to those that are fully systematized, and that is what is required. In these, as proposed by Bakeman (1978), it is fundamental to differentiate between the primary record parameters, which are the three progressive levels of frequency, order and duration (Anguera et al., 2017b). Depending on the decision of each case, which is highly relevant, and congruent with the proposed observational design, the lines to follow with regards to data quality control and data analysis are set.

#### Data Quality

In systematic observation studies, perceptual (possible unobservability, vision field reduction, etc.) and interpretative difficulties are frequently found (discretion when applying the regulation, even if it is minimal, in certain actions), and records are therefore potentially affected by various types of biases (Anguera et al., 2018a), both in inter-observers, where appropriate training is relevant, and as intra-observers, which seeks to reveal interpretative stability. Currently, the interest in complementing quantitative and qualitative approaches in the same study when carrying out data quality control has increased (Arana et al., 2016). Ample documentation on data quality exists (Blanco-Villaseñor, 1991, 1993, 2001), and specific information from each work is collected.

#### Analysis of Data and Results Obtained

From the methodological structure of observational studies, data analysis is not a capricious or unsubstantiated question, but should depend on the observational design and the nature of the data (Blanco-Villaseñor et al., 2003). There is therefore a wide range of analytical possibilities, which are basically characterized as quantitative types of analysis that stem from qualitative data, responding to the integration of qualitative and quantitative elements that characterize *mixed methods*.

## Results

### Descriptive Analysis

Of the total articles (94) analyzed, 64 are written in English (68%), 23 in Spanish (24%), and 7 in Portuguese (7%). The first article in the systematic review that gathers scientific information on observational analysis of male elite football is from 1996. After 2000, an increase in the presence of these studies is observed

Next, different aspects related to the primary works are reviewed:

#### Country of Origin of the Institutions Where Authors Belong

As for the institutions to which the authors belong, the vast majority, 54% (51) include universities in Spain (considering the participation of all universities in Spain and agreements with other institutions), making it the country with the greatest representation. Brazil is second, with 14 (15%) universities and the United Kingdom third, with 10 (10%) institutions see Table 1.

**Table 1:** Country of origin of the institutions where authors belong.

**Country**	**Frequency**	**% (*n* = 94)**
Spain	32	34.04
Brazil	11	11.70
UK	7	7.45
Spain and Portugal	7	7.45
Brasil and Portugal	3	3.19
Poland	3	3.19
Germany	2	2.13
Spain and Austria and Italy	2	2.13
Spain and France and Tunisia	2	2.13
Spain and Italy and Iceland	2	2.13
Greece	2	2.13
Norway	2	2.13
Portugal	2	2.13
Australia	1	1.06
UK and Denmark	1	1.06
Slovakia	1	1.06
Spain and Austria and UK	1	1.06
Spain and Iceland	1	1.06
Spain and Italy	1	1.06
Spain and Macedonia and Portugal	1	1.06
Spain and UK	1	1.06
Spain and USA	1	1.06
France	1	1.06
France and Italy	1	1.06
Italy	1	1.06
Italy and Sidney and Zurich and Rome	1	1.06
Japan	1	1.06
Romania	1	1.06
UK and Canada	1	1.06
USA	1	1.06

The vast majority (94.68%) of work come from universities, except for three articles that belong to a research institute (3.19%) and two others that do not specify their origin (2.12%).

#### Journals in Which Primary Studies Were Published and Impact Factor

The total number of journals in which the 94 articles were published was 36. Of this total, 14 [with 50 publications (53%)], have an impact factor, and 22 [with 44 publications (46%)], were not published in impact journals. The *Journal of Sport Sciences* presents the largest number of selected papers, with a total of 13 articles, which corresponds to 13.8% of all publications (94) and 36% of all journals (36), see Table 2.

**Table 2:** Journals in which primary studies were published and impact factor.

	**Journal**	**Quantity**	**%**	**I.F. (JCR)**.
1.	Journal of Sports Sciences	13	13.83	2.811
2.	Revista de Psicología del Deporte	6	6.38	NO
3.	Apunts. Educación Física y Deportes	6	6.38	NO
4.	European Journal of Sport Science	5	5.32	2.376
5.	International Journal of Performance Analysis in Sport	5	1.06	1.325
6.	International Journal of Sports Medicine	5	5.32	2.132
7.	Frontiers in Psychology	4	4.26	2.129
8.	Journal of Human Kinetics	5	5.32	1.414
9.	Journal of Physical Education and Sport	4	4.26	NO
10.	Journal of Sports Science and Medicine	4	1.06	1.774
11.	Perceptual and Motor Skills	3	1,06	1.049
12.	RBFF-Revista Brasileira de Futsal e Futebol	3	3.19	NO
13.	Revista Andaluza de Medicina del Deporte	3	3.19	NO
14.	Revista Brasileira de Ciências do Esporte	3	3.19	NO
15.	Cuadernos de Psicologia del Deporte	2	2.13	NO
16.	Revista Brasileira de Futebol	2	1,06	NO
17.	Procedia-Social and Behavioral Sciences	1	1.06	NO
18.	Baltic Journal of Health and Physical Activity	1	1.06	NO
19.	Cultura, Ciencia y Deporte	1	1.06	NO
20.	European Journal of Human Movement	2	2.13	NO
21.	Revista de Psicología General y Aplicada	1	1.06	NO
22.	Human Movement Science	1	1.06	1.928
23.	Physical Education and Sport	1	1.06	2.035
24.	Journal of Science and Medicine in Sport	1	1.06	3.623
25.	Journal of Sport and Health Research	1	1.06	NO
26.	Polish Journal of Sport and Tourism	1	1.06	NO
27.	Psicothema	1	1.06	1.551
28.	Revista Brasileira de Educação Física e Esporte	1	1.06	NO
29.	Revista de Preparación Física en el Fútbol	1	1.06	NO
30.	Revista Digital de Educación Física y Deportes	1	1.06	NO
31.	Revista Española de Educación Física y Deportes	1	1.06	NO
32.	Revista Internacional de Ciencias del Deporte	1	1.06	0.76
33.	Revista Mineira de Educação Física, v. Espacial	1	1.06	NO
34.	Sports	1	1.06	NO
35.	Scientific Report Series Physical Education and Sport	1	1.06	NO
36.	The Journal of Strength & Conditioning Research	1	1.06	2.49
		94	100	

#### Authors, Publication Years, Aim of Study, and Observed Events

The articles were ordered alphabetically by author, and they were assigned a numerical code, in order to facilitate their location and expedite their use. Table 3 presents authors, publication year, object of study, and observed events.

**Table 3:** Authors, publication years, object of study, and observed events.

**Number**	**References**	**Aim of the study**	**Observed events**
1	Almeida et al., 2014	The match location, status, quality of opposition on regaining possession, and zone of ball recovery.	28 Matches. Champions League. 2011–2012.
2	Andrade et al., 2012	Time of ball possession.	7 Matches. World 2010.
3	Ardá et al., 2014	Characteristics of the corner kicks and proposal of an explanatory model.	554 corner kicks performed. Along the 2010 World Cup in South Africa.
4	Armatas and Yiannakos, 2010	Characteristics of goal scoring patterns.	64 Matches. World Cup 2006.
5	Armatas et al., 2009	Characteristics of the goals scored.	240 Matches. Greek SuperLeague. 2006–07.
6	Barbosa et al., 2014	Offensive game methods, in fast attack.	12 Matches. Real Madrid 2010–2011.
7	Barreira et al., 2014	Evolution the attacking pattern.	21 national selections: 17 european teams, 3 from South America and 1 from Asian (1982–2010).
8	Barros et al., 2007	Distances covered by Brazilian soccer players and compare the results to the European players.	The trajectories of 55 players of First Brazilian Division.
9	Bradley et al., 2009	High-intensity running by soccer players.	28 Matches English Premier League 2005–2006.
10	Braz and Marcelino, 2013	Developing models for maintaining ball possession in games.	64 Matches. 2010 World Cup.
11	Buraczewski et al., 2013	Frequency and effectiveness of selected tactical and technical actions by footballers from winning and losing teams.	8 Matches. Eurocopa 2008.
12	Buscá Safont-Tria et al., 1996	Offensive individual tactics in soccer.	Michael Laudrup
13	Camerino et al., 2012a	Reveal the hidden yet stable structures which underlie the interactive situations that determine the dynamics of play.	5 National League matches and 5 Champions' League from the 2000–2001 season
14	Carey et al., 2001	The pattern of foot use in a sample of 36 players.	16 teams. 1998 World Cup.
15	Carling, 2011	The influence of opposition team formation on physical and skill-related performance.	45 Matches. French League 1; 2007–2008, 2008–2009, and 2009–2010.
16	Casáis et al., 2011	Tactical performance of winning teams and losers.	380 Matches. Spanish League 2008–2009.
17	Casal et al., 2014	The corner kicks and the efficacy.	124 Matches. 2010 World Cup (64); Eurocopa 2012 (31); Champions League 2010–2011 (29).
18	Casal et al., 2016	The efficacy of defensive transitions play in elite football.	804 Quarterbacks. World Cup 2010.
19	Casal et al., 2015a	Identify variables the corner kick and propose un model.	124 Matches. 2010 World Cup (64), Eurocopa 2012 (31), and the Champions League 2010–2011 (29).
20	Casal et al., 2017	Possession time the ball and the zone in which it develops, reflected the results the match.	12 Matches. Eighth-finals, quarterfinals, semifinals and final of the 2016 Eurocopa France.
21	Casal et al., 2015b	Predict the result of offensive transitions and the variables that intervene in them.	7 Matches. Eurocopa 2008.
22	Castañer et al., 2016	Motor skills used by Lionel Messi.	103 Goals from Messi.
23	Castañer et al., 2017	Play of Cristiano Ronaldo and Lionel Messi.	181 Goals from Lionel Messi (83) and Cristiano Ronaldo (98).
24	Castelão et al., 2015	Evaluate the offensive behaviors.	647 Matches from 6 national soccer teams participating of the finals on 2006 World Cup and 2004- and 2008-Euro Cup.
25	Castellano and Hernández-Mendo, 2000	Transitions in game action.	10 Matches. 1998 World Cup.
26	Castellano and Hernández-Mendo, 2002a	Patterns of behavior or stable transitions that exceed the probabilities marked by chance.	10 Matches. 1998 World Cup.
27	Castellano and Álvarez, 2013	Strategic use in the defense of the interaction space.	6 Matches. Spanish League. 2005-06.
28	Castellano et al., 2013	Strategic use in the use of the ball and space.	6 Matches. Spanish League 2005-06.
29	Castellano et al., 2011	Work-rate profile of a team of elite soccer.	434 Individual samples. Spanish League. 2005–06.
30	Castellano et al., 2012	Characteristics statistics between winning, drawing and losing teams.	177 Matches. 2002 World Cup (59), World 2006 (59) and World 2010 (59).
31	Castellano et al., 2009	Team interactions, collaborative strategic behaviors.	13 Matches of the 1998 World Cup and 9 Matches of the 2004 World Cup.
32	Cavalera et al., 2015	Patterns that show that changes in tertiary performance come out, may be related to changes in primary ones.	19 Matches. Italian League 2012–2013.
33	Clemente, 2012	Performance parameters and characterize the most successful teams.	208 Matches. 2010 World Cup.
34	Collet, 2013	The possession of ball in soccer.	299 Matches. Premier League, the Italian, French Ligue 1, Bundesliga, Spanish La Liga from 2007–08 to 2009–10, UEFA Champions League (2007–08 to 2009–10) and the Europa League (2009–10).
35	Di Salvo et al., 2010	Characteristics the sprinting the different playing positions.	67 matches. European Champions League and UEFA Cup (2002–2006).
36	Di Salvo et al., 2007	The independent and interactive effects of possession strategy.	30 Matches. Spanish Premier League (20) and Champions League (10).
37	Di Salvo et al., 2008	The high intensity running activity completed by elite soccer players.	62 Goalkeepers. Monitored over 109 matches. English Premier League 2003–2004 to 2005–2006.
38	Di Salvo et al., 2009	Compare match performance in professional soccer players.	563 Matches. English Premier League.
39	Fleury et al., 2009	Verify the timing of the scores and gol.	115 Matches. Brazilian Cup 2007.
40	Gómez-Ruano et al., 2012	Soccer statistics that show results related to the area.	1900 Matches. Spanish League 2003–04 to 2007–08.
41	Holienka and Farkasovsy, 2017	One-on-one game situations in football.	10 Matches. Clasificación the Eslovaquia.
42	Hughes and Franks, 2005	Comparing successful and unsuccessful teams.	64 Matches. World Cup 1990 (Italy) and FIFA World Cup 1994 (USA).
43	James et al., 2002	Areas, possession the boll, winning, draw, and losin.	21 Matches. British professional soccer (12) and European matches (9)
44	Lago-Ballesteros and Lago-Peñas, 2010	Identify specific performance indicators.	380 Matches. Spanish League 2005–2006.
45	Lago-Peñas and Anguera, 2003	Interaction between the members of a football team.	6 matches. Real Club Deportivo de A Coruña, in Spanish League and Champions League (2000–01).
46	Lago-Peñas and Lago-Ballesteros, 2011	The profiles high-intensity running.	380 Matches. Spanish League 2008–2009.
47	Lago-Peñas and Dellal, 2010	Game-related statistics allow to discriminate winning, drawing and losing teams.	380 Matches. Spanish League 2008–2009.
48	Lago-Peñas and Martín, 2007	Possession of the ball in soccer.	170 Matches. Spanish League 2008–2009.
49	Lago-Peñas and Dellal, 2010	Identify strengths that can be further developed, and weaknesses that might be improved.	380 Matches. Spanish League 2008–2009.
50	Lago-Peñas et al., 2003	The successful offensive actions in soccer.	Matches. of the Real Club Deportivo de Coruña, 2000–01.
51	Lago-Peñas et al., 2009	Effect of the location of the match, the level of the opponent and the score.	27 Matches. Spanish League 2005–2006.
52	Lago-Peñas et al., 2010	Location of the match, level of the opponent, possession of the ball and the score.	27 Matches. Spanish League, 2005–2006.
53	Lago-Peñas et al., 2011	Identify performance indicators.	288 Matches. Champions League. 2007–2008, 2008–2009, and 2009–2010,
54	Leite, 2013	Characteristics statistically the gol.	772 Matches. 19 Football World Cups from 1930 to 2010.
55	Losada, 2012	Relationship between interaction contexts and the zones marked by the position of the ball.	10 Matches. FC Barcelona (España) 2008–2009.
56	Machado et al., 2013	Characteristics the pattern in the play offensive.	28 Matches. 2010 World cup.
57	Maneiro et al., 2017a	Statistics of corner kicks.	31 Matches. Eurocopa 2012.
58	Maneiro et al., 2017b	Characteristics of corner kicks and proposal of an effective model.	64 Matches 2014 World cup.
59	Moraes et al., 2012	Characteristics of goals.	1092 goals from Brazilian Football Championship (Serie A), in 2009.
60	Njororai, 2013	The pattern and trends of the goals scored	64 Matches. World 2010.
61	Novaes de Souza et al., 2012	Tiempo de incidencia, el origen y el lugar de los goles.	380 Matches. Brasilian Championship of Football, serie A, year 2008.
62	Pino et al., 1998	Defensive action.	336 off-side situations during the European National Cup in England in 1996.
63	Planes and Anguera, 2015	Relevance of different game phases whit boll stopped.	19 Matches. FC Barcelona and Real Madrid CF in Spanish league 2011/ 2012.
64	Pollard, 2006	Geographical variations a contributing factor to home advantage.	All Matches from 52 football nations of UEFA. 1998-2003.
65	Ramos and Oliveira, 2008	Characteristics all the goals.	All goals accomplished during EuroCup 2004.
66	Rampinini et al., 2007	The influence of the opposing team, seasonal variations and the influence of first half activity the time.	34 official soccer Matches: 6 UEFA European Champions League Matches, 3 National Cup games, and 25 National League Matches.
67	Ric et al., 2016	Tactical patterns and the timescales of variables.	20 professional male soccer.
68	Sainz de Baranda and López-Riquelme, 2012	The corner kicks and the effect in the match.	64 Matches. 2006 World cup.
69	Sáinz de Baranda et al., 2011	The corner kicks and the efficacy.	64 Matches. 2006 World cup.
70	Sánchez et al., 2009	Change in point system on home advantage.	27 Seasons from 1980-1981 to 2006-2007, with 86 professional Spanish football teams from the First and Second Divisions.
71	Sánchez-Flores et al., 2012	The transcendence of the corner kick.	333 Corner throws in 35 Matches 1994 World Cup, 2010 World Cup, 2008 UEFA EURO, 2012 UEFA EURO and 2011 America Cup.
72	Santos et al., 2016	The amplitude of circulation of the ball and its variations of the corridor.	7 Matches. 2010 World cup.
73	Sarmento et al., 2011	Demonstrate the potential of the software THÈME 5.0, in the actions of counter-attack of the FC Barcelona.	12 Matches. 2009/2010 of FC Barcelona.
74	Sarmento et al., 2017	Examined the influence of tactical and situational variables on offensive sequences during elite football matches.	68 Matches. Spanish la Liga (*n* = 20 and 568), Italian Serie A (*n* = 12 and 199), German Bundesliga (*n* = 12 and 328), English Premier League (*n* = 12 and 269) and the Champions League (*n* = 12 and 330).
75	Scoulding et al., 2004	Characteristics of the Passes	6 Matches. 2002. World Cup.
76	Sgrò et al., 2016	Possession strategy, pitch location, and game period on the offensive actions performed by the winning teams.	31 Matches. Eurocopa 2012.
77	Sgrò et al., 2015	The scoring opportunities and offensive and defensive strategies.	16 Matches. Eurocopa 2012.
78	Shafizadeh et al., 2013	Characteristics of performance in successive matches for élite soccer teams from Europe.	38 Matches. Eurocopa 2012.
79	Siegle and Lames, 2012	Game interruptions of league soccer and the tactical.	16 Matches. Bundesliga.
80	Silva et al., 2009	Technical indicators that determine the performance of the teams.	All Matches. Brazilian Soccer Championship League 2008.
81	Silva et al., 2005	The behavioral patterns of the offensive process in soccer.	11 Matches. 2002 World cup.
82	Soroka, 2014	Compare the effectiveness of footballers.	31 Matches. Eurocopa and Champions League. 2012.
83	Stanculescu et al., 2014	Statistically significant parameters on the evolution soccer matches.	54 Matches: Rumanía 1ª Liga (20), Eurocopa (16), Champions League (18).
84	Szwarc, 2008	Simplified model of one-to-one play in soccer.	6 Matches. 2006 World cup.
85	Taylor et al., 2008	Match location, quality of opposition, and match status.	40 Matches of the 2002–2003 and 2003–2004 professional British football team.
86	Tenga et al., 2010a	Offensive effectiveness, such as scoring opportunities and shots at goal.	26 Matches. Spanish League 2005–2006.
87	Tenga et al., 2010b	Tactics on goal scoring by assessing opponent interactions.	163 Matches. Norwegian League 2004.
88	Thomas et al., 2006	Points system on home, advantage, tarjetas, penaltys	Red cards, yellow cards, and penalties of games played during the 2000–01, 2001–02, and 2002–03.
89	Tierney et al., 2016.	Movement patterns across the 5 most common playing formations.	11 Matches. 2014–2015 Football League season in England
90	Vigne et al., 2010	Profile of players in a top-class team.	30 home matches of a top-level Italian professional club in the 200-2005 season
91	Vogelbein et al., 2014	The time required to recover ball possession–which was operationalized as defensive reaction time.	306 Matches. Bundesliga. 2010–2011.
92	Wallace and Norton, 2014	Framework of potential causative mechanisms for patterns of play.	1966 World cup. 2010 World cup.
93	Yiannakos and Armatas, 2006	Reveal the hidden yet stable structures which underlie the interactive situations that determine the actions of attack in play.	32 Matches. Eurocopa and Champions League. 2006–2007.
94	Zurloni et al., 2014	Characteristics of goals.	19 Matches of the Italian League and Champions League. 2012–2013.

##### Authors

Of the total selected articles, 10% (9) are signed by a single author and 90% (85) are a collaboration of two or more authors.

##### Object of study

In order to find common references among the different primary works, the following terms were selected from professional football, as categories coined as such by various authors, which allowed the development of an illustrative table: goal, corner, winning, draw, losing, possession balloon, pass, home and away, distance and speed, fouls, and cards, 1 × 1 duel, penalty, marking, number of attackers, free kick, number of defenders, tactic, transition, formation.

Once the object of study is identified in each of the articles, shown in Table 4, the specific football categories observed in each study are specified.

**Table 4:** Football-specific categories that are part of the object of study in reviewed articles.

**Category**	**Articles order number**	**Quantity**	**%**
Goals	1, 4, 5, 6, 11, 12, 16, 17, 21, 22, 23, 24, 25, 29, 39, 40, 42, 43, 46, 58, 61, 63, 64, 72, 76, 84, 85, 86, 91, 92, 93.	31	32.98
Ball Possession	2, 6, 7, 10, 11, 12, 13, 15, 16, 24, 25, 26, 29, 32, 48, 50, 53, 55, 62, 63, 68, 69, 83, 85, 86, 90.	26	27.66
Corner	4, 5, 14, 16, 20, 21, 25, 27, 29, 44, 48, 53, 58, 61, 63, 64, 68, 69, 71, 77, 78, 84, 91, 92, 93.	25	26.60
Winning, draw, losing	1, 3, 17, 18, 19, 20, 21, 29, 30, 47, 48, 52, 53, 58, 61, 68, 73, 76, 77, 84, 90.	21	22.34
Passes	5, 6, 7, 14, 15, 16, 24, 44, 47, 48, 51, 55, 58, 72, 73, 77, 78, 86, 92, 93.	20	21.28
Home and away	1, 13, 16, 29, 33, 40, 41, 44, 47, 48, 51, 52, 63, 64, 90.	15	15.96
Distance and speed	8, 9, 15, 29, 35, 36, 37, 38, 45, 63, 67, 81, 87, 88, 89.	15	15.96
1x1 Duel	6, 7, 11, 12, 18, 41, 55, 61, 65, 78, 88, 92, 93.	13	13.83
Fouls and cards	16, 25, 29, 44, 48, 53, 65, 80, 83, 87.	9	9.57
Penalty	4, 5, 14, 65, 77, 88, 92, 93.	8	8.51
Free kick	14, 58, 78, 91, 92, 93.	6	6,38
Attackers	3, 17, 18, 20, 58, 73.	6	6.38
Defenders	3, 18, 20, 68, 69.	5	5.32
Classification	3, 20, 68, 69.	4	4,26
Tactics	17, 76, 74, 92.	4	4.26
Transition	19, 72, 90.	3	3.19
Formation	15, 88, 89.	3	3.19

The specific issues most studied were those related to the goal 32% (31), ball possession with 27% (26), corner with 27% (25), winning, drawing, losing with 23% (21), passes with 21% (20), Home and away and distance with 16% (15), each one; 1 × 1 duels with 14% (13), fouls and/or Red and/or yellow cards with 10% (9), penalty with 9% (8), attackers and free throws with 7% respectively (6), defenders 5% (5), Classification and tactics with 4% respectively, transition and formation with 3%, respectively. It is necessary to indicate that in almost all the primary documents, several questions are studied. The total articles in this paragraph thus exceeds 100%.

##### Item Observed events

We have considered it important to review it, although being very aware that the quantity of these do not imply a greater quality or depth of the analysis of the investigation.

In terms of the number of games studied, 24 analyzed between 1 and 20 games, 23 articles analyzed between 21 and 50 games, 21 articles analyzed between 101 and 500 games, 10 articles analyzed between 51 and 100 games and 7 articles analyzed more than 500 games. The rest of the articles analyzed isolated games or events that were not considered when selecting the number of events (hence the sum of these articles does not represent the total of the primary documents).

Four criteria can be distinguished, descending in number of articles: 1. Leagues, 2. World, 3. Other studies, and 4. Individual player analysis.

Leagues are competitions that generate the most interest in football reviewed works, with a total of 47 (50%) articles. The Champions League (15) is the most studied; followed by the Spanish League (14), the Premier League (6), La Liga Italiana (4); The French and the Bundesliga, with 3 investigations respectively; and finally an article from each of the following leagues: Brazilian, Norwegian League, Greek Super League and the League of Romania. It is necessary to clarify that more than one event could be studied in the same article, although in this study we tried to discriminate between them or explain each one separately, and therefore the sum of frequencies is not the total of the primary documents used.

World Cups occupy the second place as a study event, with a total of 30 (29%) investigations. The most studied world cup is South Africa 2010, which presents 11 articles; followed by Germany 2006 (6); France 1998 (5); continues Japan 2002 (3), and sharing last place; 1994, 2004, 1990, Italy 1990 and Brazil 2014 all with one investigation.

The third place of research interest is occupied by various sporting events, with 18 (19%) articles on the UEFA European Championships in different periods. There are also other articles that focus on games played in several world cups, seasons or championships which were followed for several years. Finally, teams from Brazil, Slovakia, Spain, and England were studied in their own nation's championships and/or in several seasons.

In fourth place there are single case studies focused on the in-depth study of an athlete, and that correspond to Messi and Laudrup, individually for each, in one case, and to Messi and Ronaldo, comparatively, in another.

### Methodological Review of the Selected Articles

According to Gough et al. (2012) and Gough (2015), we can prioritize particular characteristics. In this sense, to carry out an adequate screening we have chosen to identify the basic methodological characteristics of the published work, with acute awareness that the option for each one indicates a certain procedural positioning.

The methodological review of the primary documents has been carried out according to the aspects outlined in section *Issues for methodological review*.

Table 5 shows the 94 primary works related to the analytical review carried out. We try to show the existing procedural differences between primary documents that are manifested from the GREOM application. This allowed us to detect that statistically significant differences exist in the comparison of the following procedural aspects:

**Table 5:** Analytical review of primary works according to a GREOM adaptation (Portell et al., 2015).

**Number**	**References**	**Domain A**	**Domain B: method**					**Domain C: results**	**Comments**
		**Observational method**	**Observ. design**	**Instrument**		**Record parameter**	**Quality of data**	**Data analysis and results**	
				**Observation**	**Recording**				
1	Almeida et al., 2014	Direct observation	Not	FF/CS [Field format combined with category systems]	MATCH VISION STUDIO	Freq	Kappa	Descriptive analysis Multivariate logistic regression	Criteria are called variables. They use independent and dependent variables.
2	Andrade et al., 2012	Direct observation	Not	CS [Category System]	Video (from TV)	Freq	Kappa	Descriptive analysis Shapiro-Wilk test Mean comparation	
3	Ardá et al., 2014	Direct observation	N/S/M	FF/SC	Video (from TV)	Freq	Kappa Consensual agreement	Descriptive analysis Chi-square Logistic regression (SPSS)	Dimensions are called variables
4	Armatas and Yiannakos, 2010	Direct observation	Not	Scoreboard	Video SPORTSCOURT	Freq	Kappa	Descriptive analysis Chi-square	
5	Armatas et al., 2009	Direct observation	Not	Scoreboard	Video SPORTSCOURT	Freq	Kappa Correlation coefficient	Descriptive analysis Chi-square	
6	Barbosa et al., 2014	Direct observation	I/S/M	FF/CS	SDIS-GSEQ	Order	Kappa Consensual agreement	Lag sequential analysis	
7	Barreira et al., 2014	Direct observation	N/S/M	FF/CS	SoccerEye	Order	Kappa	Lag sequential analysis	
8	Barros et al., 2007	Direct observation	Not	Distances	Automated tracking system	Freq	Not	Descriptive analysis Kruskal-Wallis test ANOVA	
9	Bradley et al., 2009	Direct observation	Not	Categories (Does not meet the category system requirements)	Computerized tracking system (Stadium Manager, ProZone)	Freq	Variation coefficient	Descriptive analysis Normality test ANOVA Tukey's *post-hoc* tests	
10	Braz and Marcelino, 2013	Indirect observation	Not	Zonas	Video Excel	Freq Duration	Not	Descriptive analysis D'Agostino-Pearson test ANOVA Tukey test	Data downloaded from the web http://pt.fifa.com/index.html
11	Buraczewski et al., 2013	Direct observation	Not	CS	Not	Freq	Not	Descriptive analysis Mean comparison	
12	Buscá Safont-Tria et al., 1996	Indirect observation	Not	CB (Catalog of behaviors]	Video	Freq	Agreement coefficient	Descriptive analysis	Data provided by Televisió de Catalunya
13	Camerino et al., 2012a	Direct observation	N/P/M	FF/CS	MATCH VISION STUDIO	Order	Kappa	T-Patterns	
14	Carey et al., 2001	Direct observation	Not	CB	Video	Freq	Agreement percentage	Descriptive analysis Correlation	
15	Carling, 2011	Direct observation	Not	Categories	Computerized tracking AMISCO	Freq	Not	Descriptive analysis Normality test ANOVA Bonferroni test Effect sizes	
16	Casáis et al., 2011	Indirect observation	Not	CB	Not	Freq	Kappa	Descriptive analysis Kruskal-Wallis test Discriminant analysis	Data downloaded from the www.sdifutbol.com website Dimensions/behaviors are named variables Observation is called “notation system”
17	Casal et al., 2014	Direct observation	N/F/M	FF	NAC SPORT ELITE 42	Freq	Kappa	Descriptive analysis Chi-square Logistic regression	
18	Casal et al., 2016	Direct observation	N/F/M	FF/CS	NAC SPORT ELITE 42 and LINCE	Freq	Kappa	Descriptive analysis Chi-square Logistic regression	Dimensions are called variables
19	Casal et al., 2015a	Direct observation	N/S/M	FF/CS	Video	Freq	Kappa	Descriptive analysis Chi-square Logistic regression	Dimensions are called variables
20	Casal et al., 2017	Direct observation	Not	FF/CS	Video	Freq Duration	Kappa	Kruskal-Wallis test Welch test Logistic regression	
21	Casal et al., 2015b	Direct observation	N/S/M	FF	NAC SPORT ELITE 42	Freq	Kappa	Chi-square Logistic regression	Dimensions are called variables
22	Castañer et al., 2016	Direct observation	I/F/M	FC/SC (OSMOS)	LINCE	Order	Kappa	Proportions comparison Lag sequential analysos Polar coordinates	
23	Castañer et al., 2017	Direct observation	N/F/M	FC/SC (OSMOS)	LINCE	Order	Kappa	T-Patterns detection Polar coordinates	
24	Castelão et al., 2015	Direct observation	Not	CB	Video (from TV) Excel SDIS-GSEQ	Order	Not	Descriptive analysis Lag sequential analysis (SDIS-GSEQ)	
25	Castellano and Hernández-Mendo, 2000	Direct observation	Lag-log	FC/SC	Video SDIS-GSEQ	Order	Kappa Consensual agreement Kendall Pearson Spearman TG	Lag seqüencial analysis (SDIS-GSEQ)	
26	Castellano and Hernández-Mendo, 2002a	Direct observation	Lag-log	FC/SC (SOCCAF)	Video	Order	Kappa Correl. TG	TG Lag sequential analysis (SDIS-GSEQ)	
27	Castellano and Álvarez, 2013	Direct observation	Not	CB Distances	Video EXCEL Computerized tracking AMISCO	Freq	Not	Descriptive analysis Kolmogorov-Smirnov test Levene test Lineal regression Correlations	Dimensions/behaviors are named variables They use independent and dependent variables
28	Castellano et al., 2013	Direct observation	Not	Distances	AMISCO Pro	Freq	Kappa	Descriptive analysis Mean comparison ANOVA Bonferroni test Effect size	Dimension “distance” is called “variable” Semiautomatic registration System
29	Castellano et al., 2011	Direct observation	Not	Distances and CS	Amisco	Freq	Not	Descriptive analysis Mauchly's test of sphericity General linear model Repeated measures ANOVA	
30	Castellano et al., 2012	Indirect observation	Not	CS		Freq	Kappa	Descriptive analysis Dunnett *post-hoc* test Discriminant analysis	Data downloaded from the web: http://fifa.com/worldcup/index.html Behaviors are named indicators Dimensions/criteria are named variables
31	Castellano et al., 2009	Direct observation	Not	CS	Video EXCEL	Freq	Consensual agreement	Multidimensional scaling Correspondence analysis	
32	Cavalera et al., 2015	Direct observation	N/F/M	FC/SC	Video LINCE	Order	Kappa	T-Patterns	
33	Clemente, 2012	Indirect observation	Not	CB	Not	Freq	Not	Descriptive analysis ANOVA Kolmogorov-Smirnov test Levene test	Data downloaded from the web (http://www.fifa.com/worldcup/archive/southafrica2010/index.html) Observation is called “notation analysis” Behaviors are named variables, they also call them indicators The terms independent and dependent variable are used
34	Collet, 2013	Indirect observation	Not	CS Duration	Video STATA	Freq Duration	Not	Odds ratio Correlation Logistic regression	Some data was downloaded from websites: http://www.uefa.com/memberassociations/uefarankings/club/index.html http://www.fifa.com/worldranking/rankingtable/index.html
35	Di Salvo et al., 2010	Direct observation	Not	CB	Semi-automated image recognition system (Prozone^®^)	Freq	Not	Descriptive analysis Mean comparison Size effect	
36	Di Salvo et al., 2007	Direct observation	Not	Categories Rating scale Distances	Computerized tracking AMISCO	Freq	Not	Descriptive analysis ANOVA Tukey test	
37	Di Salvo et al., 2008	Direct observation	Not	Rating scale	Video	Freq	Not	Descriptive analysis Pearson correlation	
38	Di Salvo et al., 2009	Direct observation	Not	Distances and some behaviors	Semi-automated image recognition system (Prozone ®)	Freq	Coefficient of variation	Mixed linear modeling Bonferroni test	
39	Fleury et al., 2009	Indirect observation	Not	Indicators	Excel	Freq	Not	Descriptive analysis	Data downloaded from the web http://www.cbf.com.br
40	Gómez-Ruano et al., 2012	Indirect observation	Not	CB	Not	Freq	Kappa	Mixed linear model Factor analysis (using principal components and varimax rotation)	Data downloaded from the web www.sdifutbol.com They use independent and dependent variables Observation is called “notation system”
41	Holienka and Farkasovsy, 2017	Direct observation	Not	CB	Not	Freq	Not	Binomial test Mann-Whitney test	Authors consider it indirect observation
42	Hughes and Franks, 2005	Direct observation	Not	CB Scoreboard	Video	Freq	Percentage agreement	Mean comparison Regression analysis	Observation is called “notation system”
43	James et al., 2002	Direct observation	Not	CB Scoreboard	Video computerized System (Noldus Observer Video Pro)	Freq	Agreement percent	Chi-square	
44	Lago-Ballesteros and Lago-Peñas, 2010	Indirect observation	Not	CB	Not	Freq	Kappa	Descriptive analysis ANOVA	Data downloaded from the web www.sdifutbol.com Dimensions/behaviors are named variables
45	Lago-Peñas and Anguera, 2003	Direct observation	Not	FF	Video	Order	Consensual agreement Kappa	Lag sequential analysis	
46	Lago-Peñas and Lago-Ballesteros, 2011	Indirect observation	Not	BC	Not	Freq	Kappa	Descriptive analysis Kolmogorov-Smirnov test Mean comparison (t and Mann-Whitney) Structural coefficients	Dimensions/behaviors are named variables Observation is called “notation system”
47	Lago-Peñas et al., 2010	Indirect observation	Not	BC	Not	Freq	Kappa	Descriptive analysis Kruskal-Wallis test Chi-square Discriminant analysis	Data downloaded from the web www.sdifutbol.com Dimensions/behaviors are named variables Observation is called “notation system”
48	Lago-Peñas and Martín, 2007	Indirect observation	Not	BC	Not	Freq and duration	Not	Determination coefficient Regression analysis	They use independent and dependent variables Observation is called “notation system”
49	Lago-Peñas and Dellal, 2010	Indirect observation	Not	BC	Not	Freq	Kappa	Descriptive analysis Variation coefficient Regression analysis	They use independent and dependent variables Dimensions/behaviors are named variables
50	Lago-Peñas et al., 2003	Direct observation	I/F/M	FF	Video SDIS-GSEQ	Order	Kappa Consensual agreement	Descriptive analysis Lag sequential analysis	
51	Lago-Peñas et al., 2009	Direct observation	Not	Categories Rating scales	Computerized tracking AMISCO	Freq	Kappa	Descriptive analysis Lineal regression	Dimensions/behaviors are named variables They use independent and dependent variables
52	Lago-Peñas et al., 2010	Direct observation	Not	Categories	Computerized tracking AMISCO	Freq	Not	Descriptive analysis Lineal regression	Dimensions/behaviors are named variables
53	Lago-Peñas et al., 2011	Indirect observation	Not	BC	Not	Freq	Kappa	Descriptive analysis ANOVA Discriminant analysis Structural coefficients	Criteria are named variables
54	Leite, 2013	Indirect observation	Not	Scoreboard	Archive data	Freq	Not	Descriptive analysis	Data downloaded from the web www.fifa.com
55	Losada, 2012	Direct observation	N/F/M	FC/SC	MATCH VISION STUDIO	Freq	Kappa	Descriptive analysis ANOVA Log-linear analysis Correspondence analysis	
56	Machado et al., 2013	Direct observation	N/F/M	FF/SC	SoccerEye	Order	Kappa	Lag sequential analysis	
57	Maneiro et al., 2017a	Direct observation	N/S/M	FC/SC	Video	Freq	Kappa	Descriptive analysis Chi-square	Dimensions are named variables
58	Maneiro et al., 2017b	Direct observation	N/S/M	FC/SC	Video	Freq	Kappa	Descriptive analysis Chi-square Logistic regression	
59	Moraes et al., 2012	Indirect observation	Not	Categories	Video	Freq Duration	Kappa	Descriptive analysis Chi-square	Data provided by Central Digital de Dados–GFPA Dimensions are named variables
60	Njororai, 2013	Indirect observation	Not		Archive data	Freq	Kappa	Descriptive analysis	Data downloaded from the web http://www.fifa.com/worldcup/statistics
61	Novaes de Souza et al., 2012	Indirect observation	Not	Categories	Video	Freq	Third observer when disagreement existed	Kolmogorov-Smirnov test Levene test ANOVA Newman-Keuls test Friedman test	Data downloaded from the webs: Esporte.uol.com.br/futebol/campeonatos/brasileiro/, globoesporte.globo.com, www.youtube.com
62	Pino et al., 1998	Direct observation	Not	SC Rating scale	Video	Freq	Correlation coefficient	Descriptive analysis Chi-square	
63	Planes and Anguera, 2015	Direct observation	N/F/M	FF/SC	MOTS LINCE	Order	Kappa	Comparison proportions	
64	Pollard, 2006	Indirect observation	Not	Scoreboard	Not	Freq	Not	Descriptive analysis Regression analysis	Data downloaded from the webs: www.soccerway.com, www.rsssf.com and the Rothmans Football Yearbook independent and dependent variables are used
65	Ramos and Oliveira, 2008	Direct observation	Not	BC	Video	Freq	Not	Descriptive analysis	
66	Rampinini et al., 2007	Direct observation	Not	Categories	Video Semiautomatic system PROZONE	Freq Duration	Not	Descriptive analysis Normality test Sphericity test ANOVA Bonferroni post-ho test	
67	Ric et al., 2016	Direct observation	Not	Categories	SPI Pro GPSports	Freq Duration	Not	Hierarchical principal components Kruskal-Wallis test	
68	Sainz de Baranda and López-Riquelme, 2012	Direct observation	Not	CS	Video DARTFISH TEAM PRO	Freq	Kappa	Descriptive analysis Chi-square Phi coefficient Cramer statistic	Observation is also called “notation system”
69	Sáinz de Baranda et al., 2011	Direct observation	Not	CS	Not	Freq	Kappa Correlation coefficient	Descriptive analysis Chi-square	
70	Sánchez et al., 2009	Indirect observation	Not	Scoreboard	archived data	Freq	Not	Mean comparation ANOVA	Data downloaded from the web www.lfp.es
71	Sánchez-Flores et al., 2012	Direct observation	Not	CS	Video OBSERVER	Freq	Kappa Consensual agreement	Descriptive analysis Chi-square Binomial test (Poisson)	The observation instrument consists of categories and subcategories. Interplay sequentially is implicit.
72	Santos et al., 2016	Direct observation	Not	CS	Video Excel	Freq	Kappa	Descriptive analysis Kolmogorov-Smirnov test Kruskal-Wallis test	
73	Sarmento et al., 2011	Direct observation	Not	FF	Not	Order	Inter- and intra-agreement	T-Patterns	
74	Sarmento et al., 2017	Direct observation	Not	CS	Video	Freq	Kappa	Chi-square Logistic regression	
75	Scoulding et al., 2004	Direct observation	Not	CS	Video NOLDUS OBSERVER VIDEO PRO	Freq Duration	Agreement	Chi-square Mann-Whitney test	Observation is called “notational system”
76	Sgrò et al., 2016	Direct observation	Not	Indicators	Video LONGOMATCH	Freq	ICC coefficient	OTP Ratio ANOVA Logistic regression	Behaviors are named indicators
77	Sgrò et al., 2015	Direct observation	Not	BC	Video LONGOMATCH	Freq	Correlation coefficient	Descriptive analysos Shapiro-Wilk test Kruskal-Wallis test Discriminant analysis	Observation is called “notation system” Behaviors are named indicators The denomination of independent and dependent variables is used
78	Shafizadeh et al., 2013	Direct observation	Not	BC Technical data	Video SPORTS PERFORMER SOFTWARE	Freq	Not	Time-series analysis	Behaviors are named indicators
79	Siegle and Lames, 2012	Direct observation	Not	FF/CS	Video	Freq Duration	Kappa Correlation coefficient	Descriptive analysis ANOVA MANOVA	
80	Silva et al., 2009	Indirect observation	Not	BC Distances	archived data	Freq	Not	Spearman correlation	Behaviors to be observed are called ‘technical indicators’ Data downloaded from the web: www.globo.com/globoesporte
81	Silva et al., 2005	Direct observation	N/F/M	BC	Video SDIS-GSEQ	Order	Kappa	Lag sequential analysis	
82	Soroka, 2014	Direct observation	Not	BC	Video Semiautomatic system FIFA's Castrol Performance	Freq	Not	Efficacy index Efficiency index Descriptive analysis Mean comparison	
83	Stanculescu et al., 2014	Direct observation	Not	Categories	Video	Freq	Not	Descriptive analysis Means comparation	Observation is called “notation system” Behaviors to be observed are called “parameters”
84	Szwarc, 2008	Direct observation	Not	BC	Video	Freq	Not	Descriptive analysis	
85	Taylor et al., 2008	Direct observation	Not	Categories	Video NOLDUS OBSERVER VIDEO PRO	Freq	Agreement coefficient	Log-linear analysis Logit analysis	Observation is called “notation system”
86	Tenga et al., 2010c	Direct observation	Not	BC	Not	Freq	Not	Descriptive analysis Logistic regression	Criteria/behaviors are named variables Control sample was used
87	Tenga et al., 2010b	Direct observation	Not	BC	Video FINAL CUT PRO	Freq	Kappa	Descriptive analysis Logistic regression ROC curve	Dimensions/behaviors are named variables They use independent and dependent variables
88	Thomas et al., 2006	Indirect observation	Not	Rating scale	Not	Freq	Not	Descriptive analysis Chi-square	Data downloaded from the web: www.soccerbase.com
89	Tierney et al., 2016)	Direct observation	Not	BC	Not	Freq	Not	Descriptive analysis MANOVA	It is combined with an experimental method
90	Vigne et al., 2010	Direct observation	Not	BC	Video	Freq	Kappa	Descriptive analysis ANOVA	
91	Vogelbein et al., 2014	Direct observation	Not	BC Scoreboard	German PAL MathBall software	Freq and duration	Kappa α de Cronbach	Descriptive statistics Kruskal-Wallis test Mann-Whitney test Point-biserial correlation	
92	Wallace and Norton, 2014	Direct observation	Not	FF/CS	SportsTec Australia	Freq Duration	Inter e intra (binomial test)	Regression analysis Trend analysis	Also, it is a correlational study Observation is called “notation system”
93	Yiannakos and Armatas, 2006	Direct observation	Not	BC Distances	Video SPORTSCOURT	Freq	Correlation coefficient	Descriptive analysis Chi-square	
94	Zurloni et al., 2014	Direct observation	N/P/M	BC	LINCE	Order	Kappa	T-Patterns	

(a) Directobservation vs.indirect observation,(b) observational design(IPU: Idiographic/Point/Unidimensional, NPU: Nomothetic/Point/Unidimensional, IFU: Idiographic/Follow-up/Unidimensional,NFU: Nomothetic/Follow-up/Unidimensional,IPM: Idiographic/Point/Multidimensional,NPM: Nomothetic/Point/Multidimensional,IFM: Idiographic/Follow-up/Multidimensional,NFM: Nomothetic/Follow-up/Multidimensional)vs. no observational design, (c) frequency vs. order parameter, (d) data quality control vs. no data quality control, (e) only descriptive analysis vs. non-descriptive data analysis, and (f) only descriptive analysis vs. detection of regularities (although this one with less statistical significance).

Additionally, comments are included regarding relevant information not included in the previous methodological structure, constructed according to the selected minimums of GREOM.

Using the 94 primary documents, a detailed comparison analysis of proportions has been made in order to know if there is a statistically significant difference between the indicators of the methodological procedure specified in GREOM, considered in Table 5 (see Table 6). Among the results obtained, the highly significant difference, compared to the initial approach of the study, between the use of direct observation vs. indirect observation stands out, as well as that of an observational design proposal compared to those who did not propose it. Similarly, among the primary works studied, there is also statistically significant differences between studies in which only the frequency parameter was used in comparison to those based on order and duration parameters. A significant difference was also found between the authors who carried quality controlled the data and those who did not. Finally, there was also a significant difference between authors who only used descriptive statistics compared to those who used other analysis techniques (although some of them also made a descriptive analysis).

**Table 6:** Proportion comparisons corresponding to quality indicators of the observational methodology.

**Ratios to compare**		***p*-value**
Direct observation 72/94 = 0.76	Indirect observation 22/94 = 0.23	<0.001
Observational design 21/94 = 0.22	Not observational design 73/94 = 0.78	<0.001
Observational instrument 54/94 = 0.57	Not observational instrument 40/94 = 0.42	0.04
Coding with software 44/94 = 0.47	Coding without software 50/94 = 0.53	0.41
Frequency parameter (only) 78/94 = 0.83	Order parameter 16/94 = 0.17	0
Control quality of data 64/94 = 0.68	Not control quality of data 30/94 = 0.32	<0.001
Only descriptive statistics 6/94 = 0.06	Data analysis (not descriptive) 74/94 = 0.79	0
Only descriptive statistics 6/94 = 0.06	Detection of regularities 15/94 = 0.16	0.03

From the results obtained, we can suggest some methodological profiles that emerge from the primary documents analyzed, which are presented in Table 7. This proposal of methodological profiles for primary articles is based on the detailed methodological revision (Table 5) and provides five possibilities that offer a gradation of methodological consistency in descending order. Except for the E (Miscellaneous) profile, they constitute procedural schemes that are of interest and can be considered as reference guides for new works in this area.

**Table 7:** Profiles that emerge from the systematic review.

**Domain A**	**Domain B: method**					**Domain C: results**	**Profiles**	**Frequency of profiles**
**Kind of observation**	**Obser. design**	**Instrument**		**Record. parameter**	**Quality of data**	**Data analysis and results**		
		**Observation**	**Recording**					
Direct	Yes	FC/SC	Some software	Frequency	Kappa	Some not descriptive analysis	A	12
Direct	Not	FC/SC	Some software	Frequency	Kappa	Some not descriptive analysis	B	26
Indirect	Not	Not		Frequency	Kappa	Some not descriptive analysis	C	14
Direct	Yes	FC/SC		Order	Kappa	Detection of regularities	D	13
Miscellaneous							E	29

## Discussion

This systematic mixed study review has been carried out in order to synthesize 92 observational studies on elite male football. It continues by first stating some aspects derived from the descriptive analysis, and then refers to the mixed method approach. Next, some aspects derived from the descriptive analysis are exposed, and then refers to *mixed method* approaches.

### Descriptive Perspective Discussion

From a descriptive perspective, the country of origin of the authors institutions, the journals in which the articles are published and their range in terms of impact factor, authors and publication year, object of study, observed events, basic football categories, and different types of events were considered as substantial aspects.

#### Country of Origin of the Institutions Where Authors Belong

Table 1 shows the country of origin of the institutions where authors belong. The country which corresponds to the majority of the institutions where authors belong is Spain (32), which represent 33.68% of the total primary documents, and with 54% participation from other institutions (51). This data has a double interpretation: on the one hand, Spain has some of the best football teams in the world and also has a large number of followers. On the other hand, the development of observational methodology is vanguard in that country, which is evident by the development carried out in the last two decades as well as the growing number of publications in scientific journals, especially in computer program construction (Hernández-Mendo et al., 2014), generalizability theory development (Blanco-Villaseñor, 2001), the application of certain quantitative analytical techniques aimed at detecting behavior patterns, such as sequential analysis of delays (Bakeman and Quera, 2011; Lapresa et al., 2013, as a sample, according Bakeman, 1978), polar coordinate analysis (Castañer et al., 2017, as a sample, following Sackett, 1980), or T-Pattern detection (Jonsson et al., 2006, as a sample, following seminal works of Magnusson, 1996, 2000, 2018), which combine the two characteristics of suitability for categorical data as well as providing relative information about the structure of regularities detected in the game from different approaches.

We also have 11 articles with Brazilian authors, occupying the second place. It is explained by the great interest that football arouses in Brazil, although publication quality is not highlighted, most are not in English nor of high impact, and it is necessary for observational methodology to be further developed.

The third country with the highest number of authors of origin is England, where there is also a great fondness for football and high interest in match analysis.

#### Journals in Which the Articles Were Published in and Impact Factor

As reflected in Table 2, the primary documents were published in 36 journals, highlighting the Journal of Sports Sciences with 12 primary documents (13% of the total), which has an impact factor of 2,539 (2016), in quartile 1.

With 32% of articles were published in impact factor journals such as the *Journal of Human Kinetics, European Journal of Sport Science, International Journal of Performance Analysis in Sport, International Journal of Sports Medicine*, Frontiers in Psychology, and all with 5% and 4% contributions, indicating between 5 and 4 articles published. A very similar percentage among all of them indicates that, although with some variation, that all journals have a very similar interest in this type of research.

Of the 36 journals in which primary documents of this research have been published, 22 have an impact factor, which represents 77% of the total. From this we interpret that these journals are interested in publishing research carried out through observational methodology.

#### Authors, Publication Year, Object of Study, and Observed Events

In terms of the number of games studied per investigation, the largest number of articles, 22, studied between 1 and 20 games, 20 articles analyzed between 21 and 50 games, 18 articles studied between 101 and 500 games, 9 articles analyzed between 51 and 100 games and 6 articles analyzed more than 500 games.

#### Football-Specific Categories That Are Part of the Object of Study in Reviewed Articles

Table 4 shows how goals occupy the central place of interest, in line with the words of Roffé et al. (2007, p. 228), “In the sport of football, the most important event that can happen during a competition is probably the goal, for any of the two opponents. It is evident that there are others (substitutions, expulsions, errors, half part) that also have a certain value, but that may not have the determining capacity of the goal in the final result.” The goal was observed in 31 investigations, in 34% of cases. The second place of interest is ball possession, which is very important in football and was studied on 26 occasions. Third, stand out corner kicks considered as strategic plays, were observed in 28 primary documents, which shows the great importance of this type of play, not only for researchers but for football in general.

On the other hand, we can see that the events that garnered the most interest are those that received most coverage through the media and those that are more often followed by soccer fans, such as football world championships, championship competitions and national leagues. Investigating at an individual level the most outstanding players called, “prodigies,” are also interesting, such as Messi, Ronaldo, or Laudrup.

#### Methodological Approach

In accordance with the *Guidelines for reporting evaluations based on observational methodology* (GREOM) (Portell et al., 2015), recognized by EQUATOR Network, the analytical review of primary documents has allowed to evidence the procedural profile of each work, from the approach of the observational methodology.

In Table 6 the methodological structure of each primary document is presented in detail, which has allowed for a procedural profile extraction, considering the A domains corresponding to the observation type (direct/indirect); B, corresponding to Method (observational design, observation and recording instrument, primary parameter of registration, and data quality control); and C, corresponding to data analysis.

From the detailed analysis of each primary document it has been possible, on the one hand, to make a proportional comparison between the compliance or lack thereof of different methodological aspects (see Table 6); and, on the other, to extract different profiles, which allow us to reflect on the *mixed methods* perspective (see Table 7).

### Systematic Mixed Study Review Discussion

According to the profile that should characterize it, the integration made between qualitative and quantitative elements stands out, as required by the *mixed method* perspective, and from a double point of view.

On the one hand, the primary documents conform to the observational methodology, and in certain cases it can be considered a mixed method in itself. In Anguera and Hernández-Mendo (2016), it is argued that the observational methodology approach, based on qualitative-quantitative complementarity, consists of an orderly succession of stages, which are: 1) obtaining qualitative data, 2) progressive transformation in a quantification record process—although the quantitizing that we propose is not used by Tashakkori and Teddlie (1998) or Sandelowski et al. (2009), 3) quantitative treatment of information from analytical techniques adequate for categorical data (Anguera et al., 2014), 4) Result interpretation with a return to qualitative data, perfectly embedded in *mixed methods*.

Classical authors in the *mixed methods* discipline, such as Creswell et al. (2003, p. 233), refer to quantification, as we have indicated in the Introduction. But the great difference with observational methodology (Anguera et al., 2017b), in favor of it, is that quantification is more robust, since it is not only based on the counting of behavior occurrences, such as frequency, but in other primary parameters (Bakeman, 1978; Anguera et al., 2001; Bakeman and Quera, 2011; Quera, 2018) such as order and duration, which present a progressive order of inclusion.

Deepening this issue further, in Anguera et al. (2017b), it is clearly argued that quantification (also known as “*quantitizing”*) in observational methodology is particularly robust, and the progressive inclusion order of the primary frequency-order-duration parameters (Bakeman, 1978) refers to the fact that frequency is the parameter that provides the least information, order adds sequence information essential for the detection of regularities (*patterns of behavior*) and knowing the structure that emerges from recorded data—and duration adds to order and frequency time units that can successfully contribute to modulating regularities and structure information.

In Anguera et al. (2017b, p. 6) it is said: “This specific consideration of the order parameter is crucial for detecting hidden structures through the quantitative analysis of relationships between different codes in systematized observational datasets (…). Precisely because it contains information on order and duration, the initial data set, which is derived from an extremely rich qualitative component, can be analyzed using a wide range of quantitative techniques, producing a set of quantitative results that are then interpreted qualitatively, permitting seamless integration.” Consequently, the order parameter is essential in this *quantitizing* that we contemplate, and that we collected in profile D of Table 7. This shows how the 13 primary documents that form it can be considered *mixed methods* themselves, by realizing the *quantitizing* using the order parameter from the registry. Furthermore, as a result of this *quantitizing* power, it is possible to perform a powerful quantitative analysis, such as the lag sequential analysis (Lago-Peñas and Anguera, 2003; Silva et al., 2005; Barbosa et al., 2013; Lapresa et al., 2013; Barreira et al., 2014) and more recently, the polar coordinates analysis (Castellano and Hernández-Mendo, 2002b; Castañer et al., 2016, 2017; Aragón et al., 2017), or T-Pattern detection (Cavalera et al., 2015; Amatria et al., 2017; Lapresa et al., 2018), all of them originating from qualitative data.

From another point of view, an integration between qualitative and quantitative elements was also made here: the qualitative review was carried out on different procedural aspects of the GREOM in all primary documents and the quantitative analysis made by the proportion comparison (Table 6) allowed us to delimit the different methodological approaches made in systematic observation studies and to quantitatively know to what extent they have been contemplated in the primary documents.

## Limitations

As in all research work, different limitations should be noted:

- Traditionally, few *mixed methods* studies have been published in the field of Sports Science, as evidenced in Table 1 of Anguera et al. (2017b). The number of studies is smaller if we focus on observational studies (Camerino et al., 2012b), with even fewer studies on elite male football.
- There is still no methodological “culture” on systematic observation in the case of some authors, and a consequence of this limitation is the high number of works in the E profile (Miscellanous), in which there are missing elements that we consider substantial for this methodology.
- We previously referred (section Journals in which primary studies were published and impact factor) that 22 of the journals that primary documents were selected from for this study have no impact factor. Although a minority in comparison to the total, we would have preferred better data.

## Conclusions

Based on a careful selection of 94 primary documents, a *systematic mixed study review* of observational studies in elite male football has been carried out. A comprehensive synthesis of evidence the aim of this study, which was reached after conducting a rigorous review of primary documents from different points of view.

A rare feature, but on which authors have placed special emphasis, is to consider this review both from the methodological approach and substantive approach.

For the systematic review carried out, the *mixed methods* perspective was adopted because it is one of the most relevant approaches on the field. This method is extremely powerful and is considered the best analysis approach for scientific studies on football. Within this approach, observational methodology is the *leitmotiv* that structures all elite male football studies, and GREOM is used as the guiding thread. Consistent with this approach, each of the different essential aspects were considered in the revision of the primary documents, and the differences between them have been assessed. Likewise, some profiles have been developed that allow methodological characterization of each of the primary documents.

Finally, we position ourselves in the mixed methods perspective, with the desire to integrate qualitative and quantitative elements in conventional systematic reviews, and have thus added the preparation of some methodological profiles which open new perspectives.

## Data Availability Statement

The datasets generated for this study are available on request to the corresponding author.

## Author Contributions

MP developed the initial idea and the project and drafted the manuscript. MA contributed to the development of project from methodological side. MO and DL contributed to development of manuscript. All authors approved the submitted version of the mansucript.

### Conflict of Interest

The authors declare that the research was conducted in the absence of any commercial or financial relationships that could be construed as a potential conflict of interest.
