# Corrigendum: Observational Studies in Male Elite Football: A Systematic Mixed Study Review

**DOI:** 10.3389/fpsyg.2019.02682

**Published:** 2019-12-16

**Authors:** Maria Preciado, M. Teresa Anguera, Mauricio Olarte, Daniel Lapresa

**Affiliations:** ^1^Department of Social Psychology and Quantitative Psychology, Faculty of Psychology, University of Barcelona, Barcelona, Spain; ^2^Faculty of Psychology, Institute of Neurosciences, University of Barcelona, Barcelona, Spain; ^3^National Administrative Department of Statistics, Bogotá, Colombia; ^4^Department of Educational Sciences, University of La Rioja, Logroño, Spain

**Keywords:** professional soccer, mixed methods, systematic observation, methodological systematic review, GREOM

In the published article, there was an error in affiliation “1.” Instead of “Puigvert Foundation, Barcelona, Spain,” it should be “Department of Social Psychology and Quantitative Psychology, Faculty of Psychology, University of Barcelona, Barcelona, Spain.”

Due to the above change, a correction has also been made to the **Acknowledgements** section:

“The authors gratefully acknowledge the support of a Spanish government subproject *Integration ways between qualitative and quantitative data, multiple case development, and synthesis review as main axis for an innovative future in physical activity and sports research* [PGC2018-098742-B-C31] (2019–2021) (Ministerio de Ciencia, Innovación y Universidades, Programa Estatal de Generación de Conocimiento y Fortalecimiento Científico y Tecnológico del Sistema I+D+i), that is part of the coordinated project *New approach of research in physical activity and sport from mixed methods perspective* (NARPAS_MM) [SPGC201800X098742CV0]. In addition, the second author thanks the support of the Generalitat de Catalunya Research Group, *GRUP DE RECERCA I INNOVACIÓ EN DISSENYS (GRID). Tecnolog*í*a i aplicació multimedia i digital als dissenys observacionals* [Grant number 2017 SGR 1405]. Also, this study was funded by grants from the University of La Rioja. MP also recognizes the support of the University of Barcelona (Vice-Rectorate for Doctorate and Research Promotion) for the funds it obtained.”

The authors apologize for this error and state that this does not change the scientific conclusions of the article in any way. The original article has been updated.

